# Stomach Volvulus Combined With Small Bowel Obstruction Eight Years After Laparoscopic Gastric Plication: A Case Report

**DOI:** 10.7759/cureus.118504

**Published:** 2026-10-24

**Authors:** Nikolaos Pararas, Anastasia Pikouli, Despoina Papantoniou, Dionysios Dellaportas, Emmanouil Pikoulis

**Affiliations:** 1 3rd Department of Surgery, Attikon University Hospital, National and Kapodistrian University of Athens, Athens, GRC

**Keywords:** bariatric surgery and small bowel obstruction, bariatric surgery complication, gastro-duodenal intussusception, laparoscopic gastric plication, laparoscopic sleeve gastrectomy (lsg), obstructive jaundice, organoaxial gastric volvulus

## Abstract

Laparoscopic gastric plication (LGP) is a restrictive bariatric procedure that reduces gastric volume without resection and without the use of staplers or prosthetic materials. We present the case of a 25-year-old female patient who had undergone LGP eight years earlier and presented with acute abdominal pain, vomiting, and recurrent episodes of obstructive jaundice. Computed tomography demonstrated gastric volvulus, and emergency laparotomy revealed a partially unfolded plicated fundus due to ruptured sutures, causing a mixed-type gastric volvulus, together with intussusception of ischemic, hypertrophic gastric mucosa from the plicated fundus through the pylorus into the duodenum, obstructing the duodenal lumen. We took down the plication, reduced the intussuscepted mucosa, and performed a sleeve gastrectomy. The postoperative course was uneventful, and the jaundice resolved. Gastric volvulus and mucosal intussusception causing obstruction are rare but potentially devastating late complications of greater curvature plication. Awareness of this spectrum of complications allows timely recognition and treatment and should be considered when choosing a bariatric procedure.

## Introduction

Obesity is a major growing health problem worldwide. Having epidemic proportions, studies estimate that more than half the global population will be living with overweight or obesity within 12 years unless prevention and treatment improve [1]. Despite advances in pharmacological treatment, bariatric surgery is the only solution for sustained weight loss and decreased comorbidity prevalence in the morbidly obese [2].

Gastric plication, also known as gastric imbrication or laparoscopic gastric plication (LGP), is a minimally invasive surgical procedure designed to aid in weight loss by reducing the size of the stomach. Folding and suturing the stomach to create pleats and folds along the greater curvature restricts stomach volume and, proportionally, the amount of food it can hold [3]. Reducing stomach volume limits how much food a person can eat, which can contribute to weight loss by decreasing caloric intake. Unlike other bariatric weight loss surgery types, LGP does not involve removing a portion of the stomach or rerouting the digestive tract. Like every other surgical procedure, postoperative complications may arise, occasionally unfamiliar to the existing literature [4]. According to the National Institute of Diabetes and Digestive and Kidney Diseases [5], the most widely performed weight-loss operations are gastric sleeve, gastric bypass, and adjustable gastric band. These operations have shown long-term sustained weight loss, and extensive literature on their complications is available. Moreover, the American Society for Metabolic and Bariatric Surgery [6] annually publishes estimates of bariatric surgery numbers, reflecting the general acceptance of sleeve gastrectomy as the most commonly performed bariatric procedure.

 This report presents the case of a patient admitted for a rare combination of LGP complications, along with its management. Following acute abdomen signs, diagnostic imaging revealed gastric volvulus and intestinal obstruction, mandating urgent complex surgical management. During exploratory laparotomy, stomach mucosa hypertrophy was identified extending up to the ampulla of Vater, obstructing the gastrointestinal lumen.

## Case presentation

Diagnostic computed tomography imaging revealed stomach volvulus on the longitudinal axis (Figure 1), and the patient was scheduled, after obtaining her informed consent, for an emergency exploratory laparotomy. The hospital’s Ethics Committee approved this study, and the patient provided informed consent to be included in the current study. A 25-year-old white female patient presented with symptoms of severe diffuse abdominal pain, vomiting, and transient obstructive jaundice for the last five months. The patient’s surgical history began with an LGP procedure eight years ago for weight loss, followed by recurrent peptic ulcer hemorrhage attributed to NSAID abuse, which was treated conservatively. Laparoscopic cholecystectomy was also performed three months prior due to symptoms that were attributed at the time to cholelithiasis/cholecystitis. Gallbladder removal did not eradicate the epigastric pain, which also radiated to the back; therefore, endoscopic retrograde cholangiopancreatography (ERCP) was performed along with stent placement for bile duct decompression, temporarily resolving the obstructive jaundice. Nevertheless, the transient attacks of obstructive jaundice continued, and the patient was also treated with antibiotics twice for recurrent cholangitis during the month before presenting to our department. Diffuse abdominal and pelvic pain prompted the patient to seek medical advice. Laboratory investigations on admission are presented in Table 1. Alkaline phosphatase (ALP), gamma-glutamyl transferase (GGT), and C-reactive protein (CRP) were elevated, whereas total bilirubin was within the normal range. The low hematocrit was attributed to chronic anemia, a well-described result of bariatric surgery [7].

**Figure 1:**
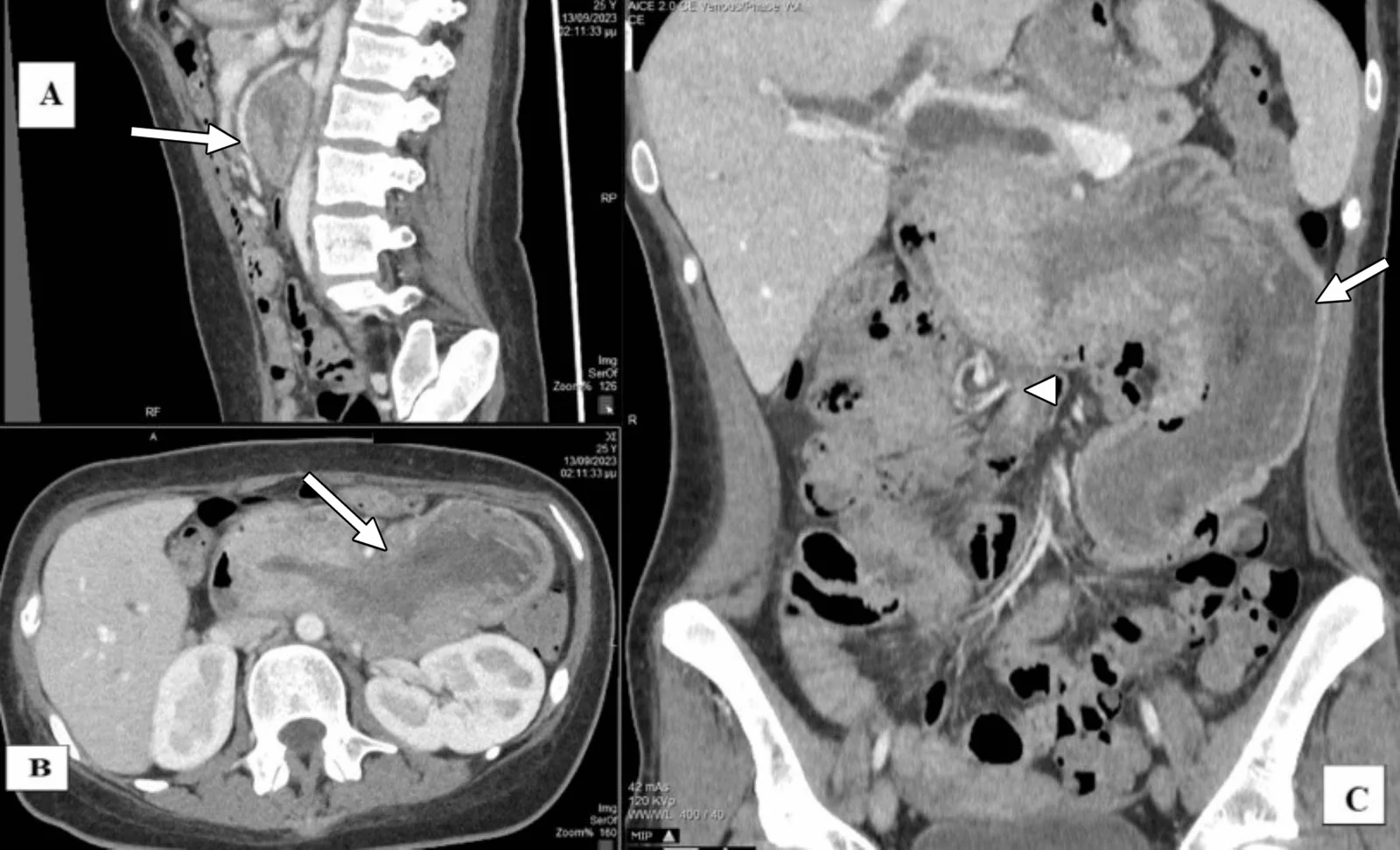
Contrast-enhanced CT Contrast-enhanced CT in sagittal (A), axial (B), and coronal (C) views showing complex twisting and turning of the stomach, not along any particular axis, consistent with a mixed/combined-type gastric volvulus.

**Table 1:** Laboratory investigations on admission †Reference ranges are those of the hospital laboratory for adult women. ALP, alkaline phosphatase; GGT, gamma-glutamyl transferase; CRP, C-reactive protein

Parameter	Patient value on admission	Reference range†
ALP	588 U/L	35–104 U/L
GGT	364 U/L	6–42 U/L
CRP	74.4 mg/L	0–5 mg/L
Total bilirubin	0.46 mg/dL	0.3–1.2 mg/dL
Hematocrit	27.10%	36–46%

During the operation, numerous adhesions extending from the fundus of the stomach to the pylorus were identified, and part of the plicated stomach was found unfolded due to ruptured sutures. The unfolded fundus created a mixed-type volvulus of the stomach not along any particular axis (Figure 2). 

**Figure 2:**
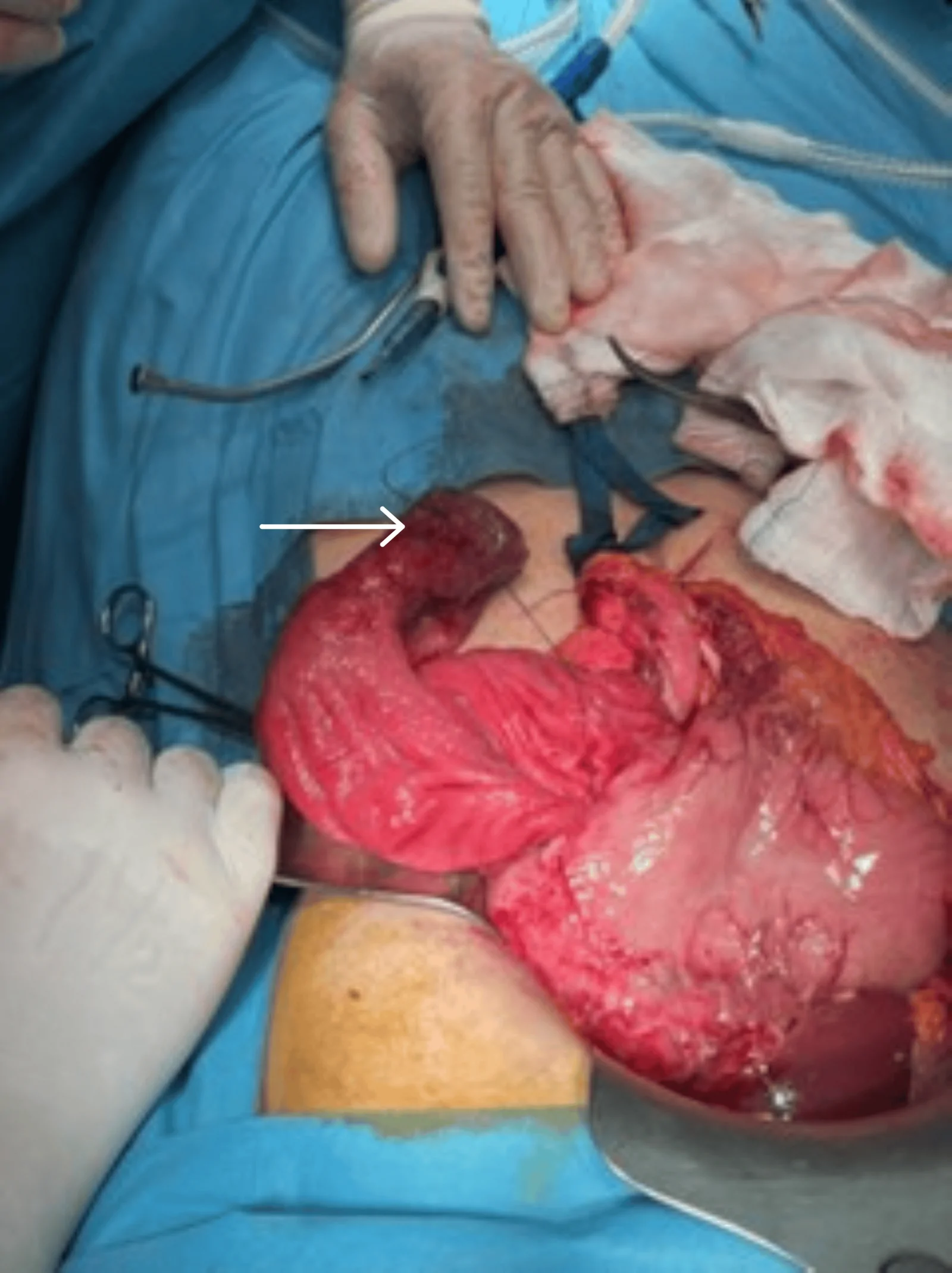
Intraoperative images Intraoperative aspect of the unfolded gastric fundus, creating gastric distention and volvulus

Moreover, a new finding not shown on the CT scan was a palpable, mobile mass originating from the stomach, passing through the pylorus, and extending to the fourth part of the duodenum. The mass was causing duodenal obstruction, which was evidently responsible for the jaundice. After adhesiolysis and restoration of normal stomach anatomy by removing all sutures from the plicated stomach, we manually reduced the mass toward the stomach. To inspect the mass, we performed a gastrotomy at the antrum and identified the mass as mucosal intussusception from the previous LGP. The mucosa originated from the previously plicated fundus of the stomach and appeared ischemic (Figures 3-6). Next, the entire greater curvature was unfolded from the previous plication, and a sleeve gastrectomy was performed. The excised part of the stomach also included the ischemic mucosa. A drainage tube was positioned along the stapler line. Methylene blue and bubble tests were performed, and they were negative for leak. The surgical wound was then sutured in the usual manner, and the patient was transferred to the surgical ward. The patient had an uneventful postoperative course; the jaundice subsided, and she tolerated oral feeding. She was discharged on the seventh postoperative day. At the three-year follow-up, the patient remained asymptomatic, with no further complications. Upper gastrointestinal endoscopy performed at two years postoperatively showed no abnormalities related to the sleeve gastrectomy.

**Figure 3:**
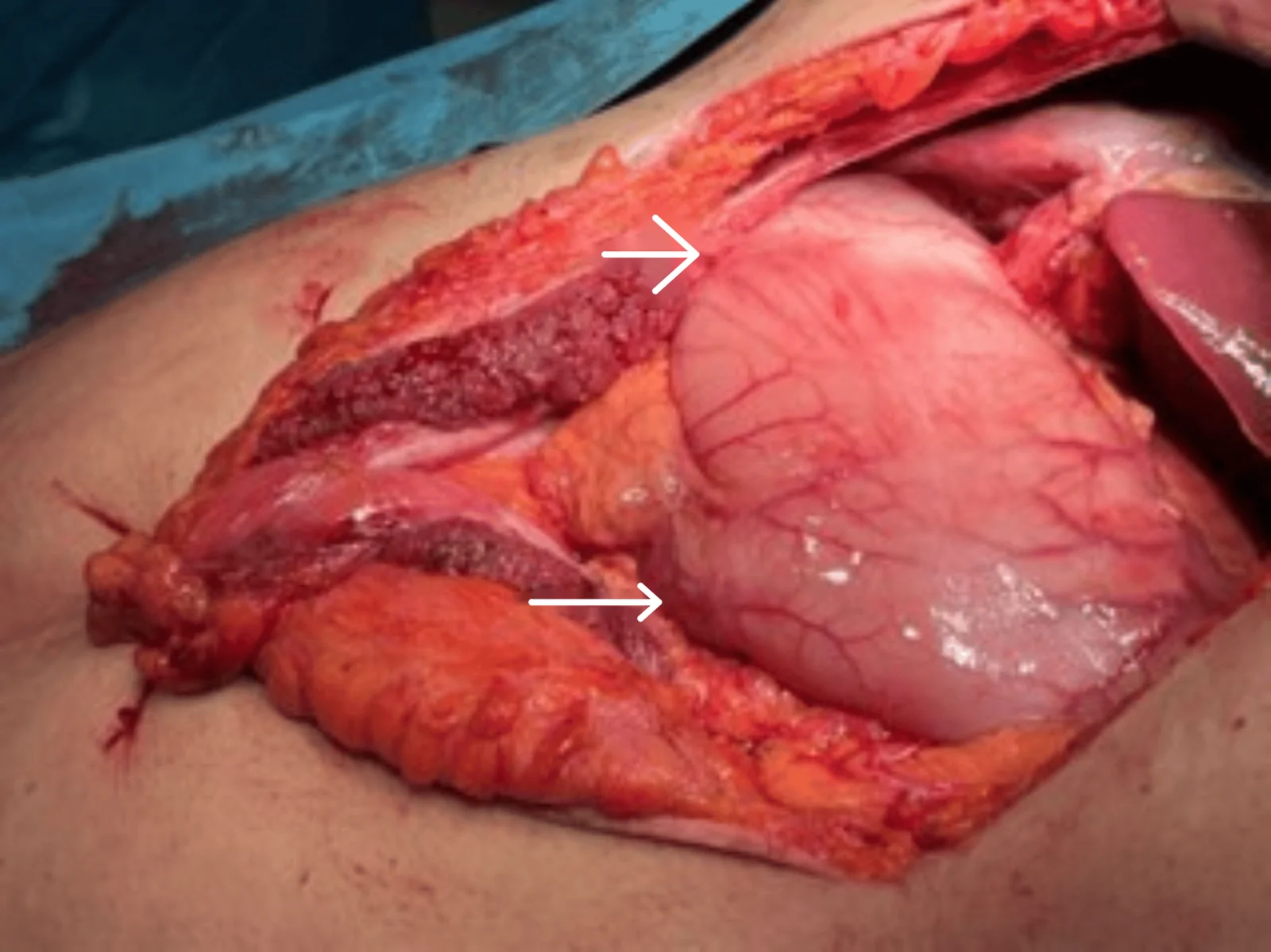
Intraoperative image The short arrow shows the pylorus, and the long arrow shows the greater curvature of the stomach.

**Figure 4:**
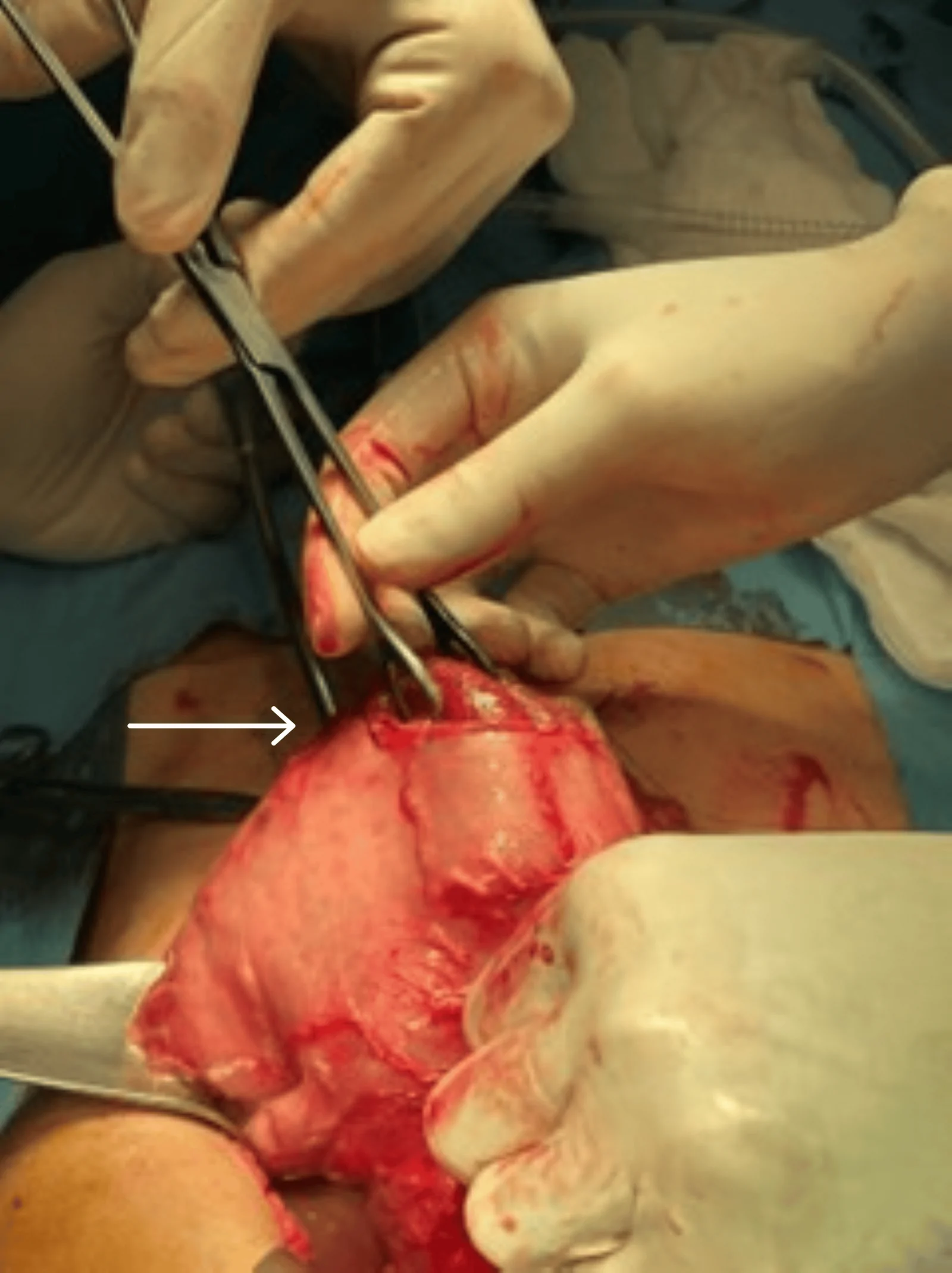
Intraoperative image Gastrotomy at the antrum of the stomach (arrow)

**Figure 5:**
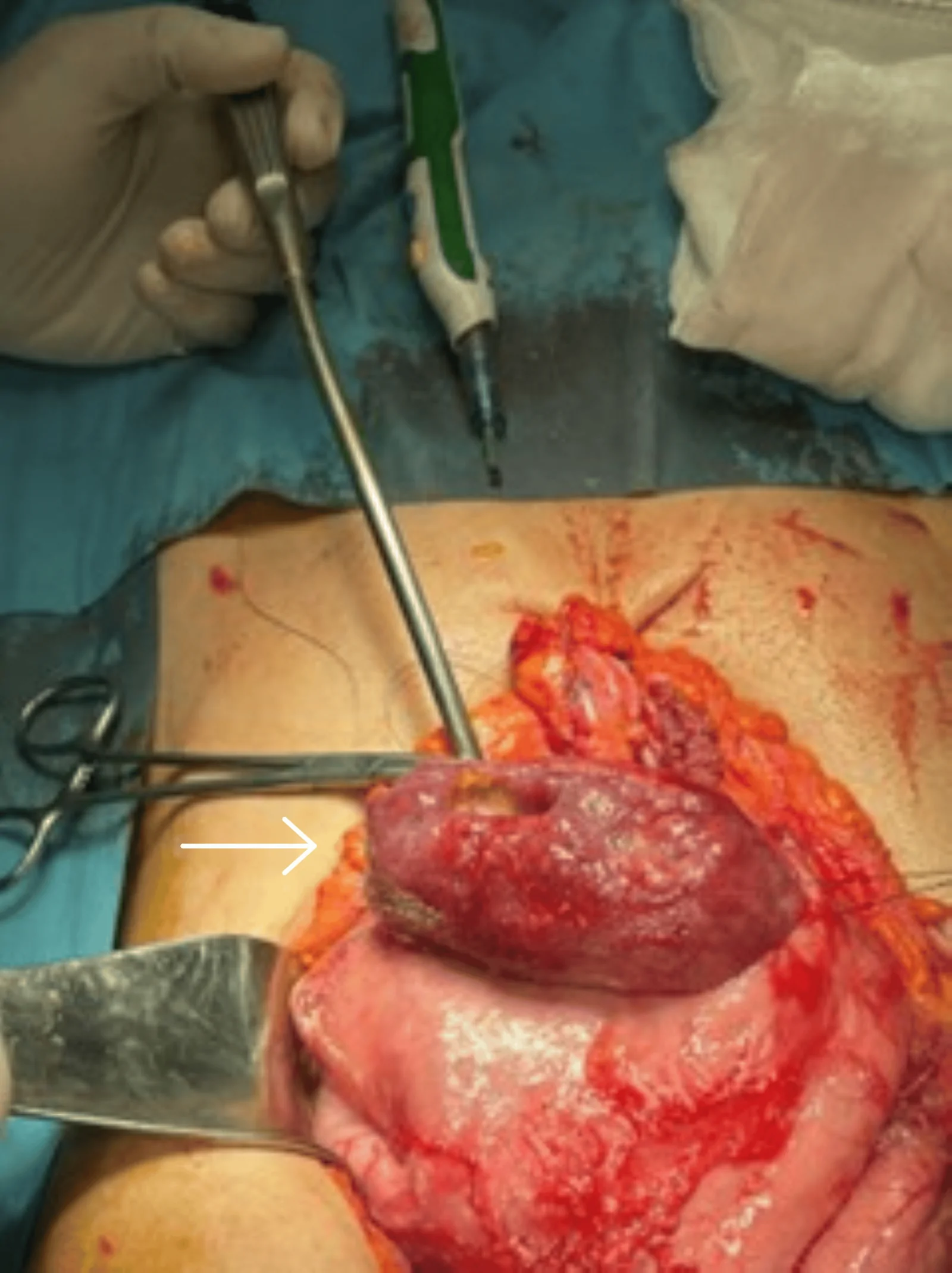
Intraoperative image Gastrotomy at the antrum of the stomach revealing the mucosal intussusception from the previous laparoscopic gastric plication (arrow)

**Figure 6:**
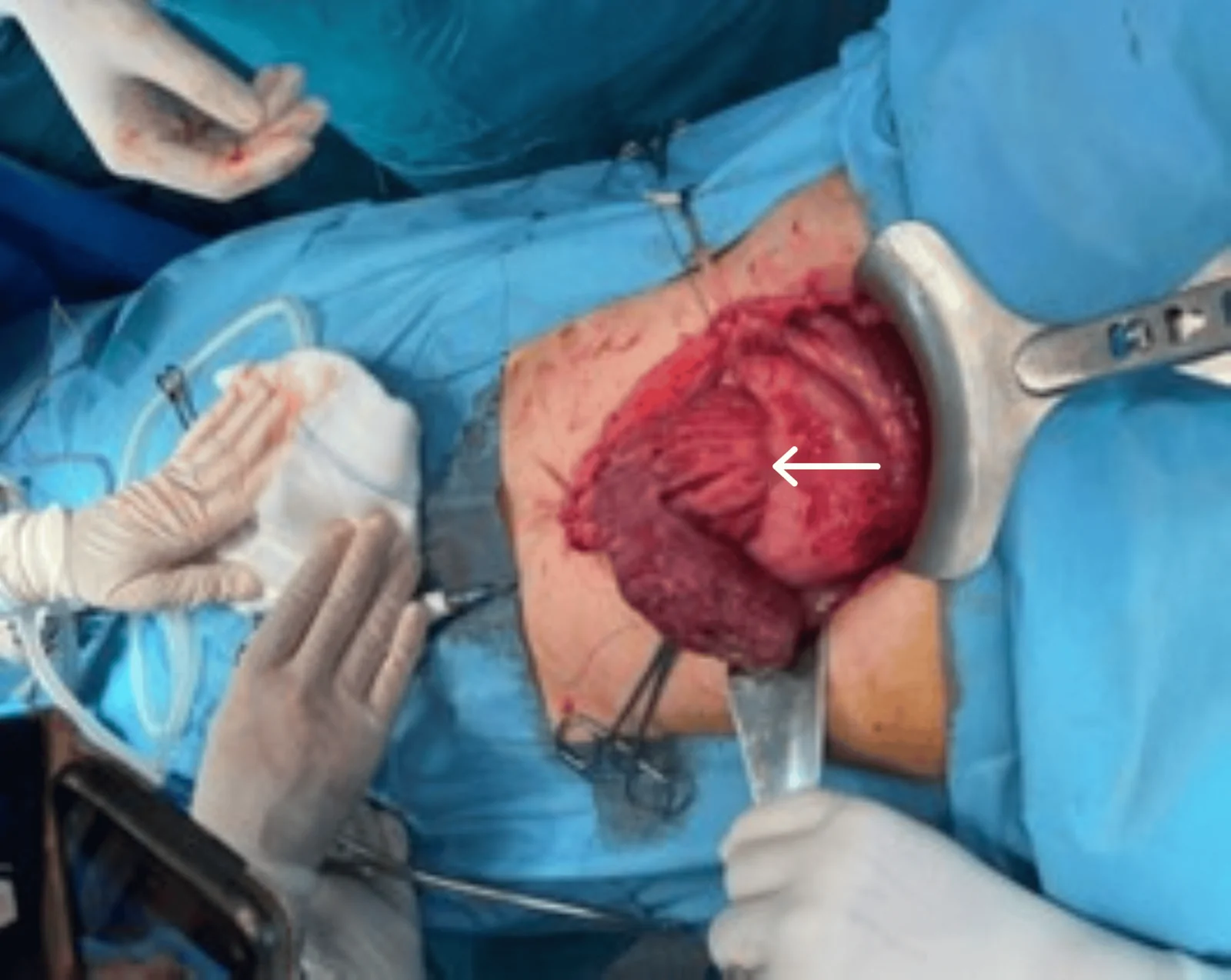
Intraoperative image Gastrotomy at the antrum of the stomach revealing the mucosal intussusception from the previous laparoscopic gastric plication. The mucosa originates from the previously plicated fundus of the stomach (arrow), which appears ischemic.

## Discussion

LGP is a constrictive type of surgical operation for morbid obesity in which no tissue is excised, and no prosthetic materials are introduced. The sutures are the sole cause of postoperative complications. The most common complications that a surgeon should be aware of are gastric remnant distention, gastric intussusception, and marginal ulcers [4].

Two surgical variations exist, differentiated by the suture placement: plication of the anterior wall of the stomach and, more commonly, greater curvature plication, which requires short gastric vessel ligation and greater curvature mobilization. The latter, which was used in the presented patient, has shown greater efficacy in weight loss [8].

However, greater curvature mobilization is associated with stomach volvulus due to insufficient tension from the greater omentum. Stomach volvulus is defined as overstretching of the stomach due to abnormal rotation. As we found in our case, the stomach can rotate 180° around its longitudinal axis, leading to bowel obstruction [9]. Timely diagnosis and immediate surgical intervention for gastropexy or, if the circumstances permit, another bariatric operation, such as sleeve gastrectomy, is of vital importance, as, if not treated, it can progress to strangulation and irreversible ischemia of the gastric tissue [10]. Kachi et al. [11] extensively reported a case of LGP complicated by gastric volvulus and its associations.

Alongside the gastric volvulus, exploratory laparotomy on our patient revealed mucosal hypertrophy extending through the pylorus and ultimately obstructing the ampulla of Vater. Almulaifi and Mohammad [12] were the first to report such an unusual complication after LGP, attributing the obstruction to gastric fold herniation. In contrast, our patient had gastric mucosal hypertrophy, leading to a solid, “plug-like” mucosal mass, without easily identifiable gastric folds.

It is worth noting that, when resolving bariatric surgery complications, if anatomically and physiologically plausible, measures should be taken to maintain the weight-loss goal. In our case, because the previously performed LGP had acceptable weight-loss results and we had to remove the fundus with ischemic hypertrophic mucosa, we decided to perform a sleeve gastrectomy to sustain weight loss [13].

In bariatric surgery, sleeve gastrectomy is considered the gold standard among restrictive methods. Therefore, it is worth comparing outcomes in patients treated with LGP with those treated with sleeve gastrectomy. The meta-analysis by Perivoliotis et al. [14] includes the most relevant studies in the field. Notably, they conclude that sleeve gastrectomy has a significantly safer postoperative profile with a smaller overall complication rate. In contrast, comparative studies on weight loss quantification have not yet been conclusive. The ideal operation for bariatric patients should combine sustained optimal weight loss, minimized complications, and reduced economic cost for candidates [15].

To the best of our knowledge, this is the first literature reference describing two previously described but rare LGP complications in a single patient. Simultaneous clinical symptoms of these two postoperative findings increase diagnostic complexity and can lead to inadequate management. Beyond detailed patient history documentation, clinical examination, and diagnostic imaging, bariatric surgery expertise enables the physician to identify such unique cases in a timely manner and act promptly to avoid catastrophic outcomes. Timely intervention and appropriate management are vital to improve outcomes and restore the patient’s safety and quality of life.

## Conclusions

This case shows that laparoscopic greater curvature plication can fail many years after surgery, when suture rupture and unfolding of the plicated fundus lead to a mixed-type gastric volvulus. In the same patient, intussusception of hypertrophic, ischemic plicated mucosa through the pylorus caused duodenal obstruction that presented as recurrent obstructive jaundice and cholangitis and was initially attributed to biliary disease. Computed tomography identified the volvulus but not the mucosal intussusception, which was recognized only at laparotomy. Taking down the plication, reducing the intussuscepted mucosa, and converting to sleeve gastrectomy relieved obstruction and resolved the jaundice while preserving weight loss.

## References

[1] Lobstein T, Jackson-Leach R, Powis J, Brinsden H, Gray M (2026). World Obesity Atlas 2023. World Obesity Atlas.

[2] Sjöström L, Lindroos AK, Peltonen M (2004). Lifestyle, diabetes, and cardiovascular risk factors 10 years after bariatric surgery. N Engl J Med.

[3] Kourkoulos M, Giorgakis E, Kokkinos C, Mavromatis T, Griniatsos J, Nikiteas N, Tsigris C (2012). Laparoscopic gastric plication for the treatment of morbid obesity: a review. Minim Invasive Surg.

[4] Khorgami Z, Ortiz Lagardere A (2018). Complications of gastric plication. Prevention and Management of Complications in Bariatric Surgery.

[5] (2026). Types of Weight-loss Surgery. https://www.niddk.nih.gov/health-information/weight-management/bariatric-surgery/types.

[6] (2026). Estimate of Bariatric Surgery Numbers, 2011-2023. Gainesville (FL): ASMBS.

[7] Gowanlock Z, Lezhanska A, Conroy M, Crowther M, Tiboni M, Mbuagbaw L, Siegal DM (2020). Iron deficiency following bariatric surgery: a retrospective cohort study. Blood Adv.

[8] Brethauer SA, Harris JL, Kroh M, Schauer PR (2011). Laparoscopic gastric plication for treatment of severe obesity. Surg Obes Relat Dis.

[9] Rashid F, Thangarajah T, Mulvey D, Larvin M, Iftikhar SY (2010). A review article on gastric volvulus: a challenge to diagnosis and management. Int J Surg.

[10] Zafar M, Parvin J, Mcwhirter A (2022). Gastric volvulus: diagnosis and successful endoscopic de-rotation towards conservative management in a patient with multiple comorbidities. Cureus.

[11] Kachi A, Chidiac K, Khaled C (2020). A rare case of gastric volvulus post laparoscopic gastric greater curvature plication. Surg J (N Y).

[12] Almulaifi A, Mohammad WM (2013). Obstructive jaundice: a rare complication of laparoscopic greater curvature plication. J Surg Case Rep.

[13] Sepúlveda M, Alamo M, Saba J, Astorga C, Lynch R, Guzmán H (2017). Long-term weight loss in laparoscopic sleeve gastrectomy. Surg Obes Relat Dis.

[14] Perivoliotis K, Sioka E, Katsogridaki G, Zacharoulis D (2018). Laparoscopic gastric plication versus laparoscopic sleeve gastrectomy: an up-to-date systematic review and meta-analysis. J Obes.

[15] Najjari K, Fattahi MR, Bariklou A (2024). Laparoscopic gastric plication: a long-term follow-up and comparison of outcomes in severe vs. non-severe obesity. Am J Surg.

